# Risk factors for femoral overgrowth after femoral shortening osteotomy in children with developmental dysplasia of the hip

**DOI:** 10.3389/fped.2023.1104014

**Published:** 2023-03-09

**Authors:** Haotian Pang, Ruoyi Guo, Hanjie Zhuang, Yulong Ben, Yue Lou, Pengfei Zheng

**Affiliations:** Department of Orthopaedic Surgery, Children’s Hospital of Nanjing Medical University, Nanjing, China

**Keywords:** developmental dysplasia of the hip, femoral shortening osteotomy, overgrowth, leg length discrepancy, children

## Abstract

**Objective:**

Developmental dysplasia of the hip (DDH) refers to a series of deformity of acetabulum and proximal femur and abnormal relationship between them, it represents the most common hip disease in children. Overgrowth and limb length discrepancy (LLD) was common complication in children undergoing femoral shortening osteotomy. Therefore, the purpose of this study was to explore the risk factors of overgrowth after femoral shortening osteotomy in children with DDH.

**Methods:**

We included 52 children with unilateral DDH who underwent pelvic osteotomy combined with femoral shortening osteotomy between January 2016 and April 2018, including seven males (six left and one right hip) and 45 females (33 left and 12 right hips) with an average age of 5.00 ± 2.48 years, and an average follow-up time of 45.85 ± 6.22 months. The amount of overgrowth and limb length discrepancies (LLDs) were calculated. The risk factors of femoral overgrowth ≥1 cm and LLD ≥ 1 cm were analyzed.

**Results:**

There were statistical differences in age (*p *< 0.001) and operation duration (*p *= 0.010) between the two groups with femoral overgrowth <1 cm and ≥1 cm. There was a statistical difference in operation duration (*p *< 0.001) between the two groups. Age (*p *< 0.001) was an independent influencing factor of femoral overgrowth in children with unilateral DDH after pelvic osteotomy and femoral shortening osteotomy, and a risk factor (*p *= 0.008) of LLD in these children.

**Conclusion:**

The overgrowth and LLD of children with developmental dislocation of hip after pelvic osteotomy and femoral shortening osteotomy are significantly related to age. There was no significant difference between different pelvic osteotomies for femoral overgrowth in children. Therefore, surgeons should consider the possibility of LLD after femoral shortening osteotomy in children of a young age.

## Introduction

Developmental dysplasia of the hip (DDH) is a common deformity of the lower limb in children (1). Tönnis described a pelvic radiographic classification of DDH to show the dislocation height of the femoral head, which depending on the location relationship between the ossification of the femoral head and the acetabulum. Tönnis grade I refers to the ossification center of the capital epiphysis is medial to the perpendicular line from the superolateral margin of the acetabulum (Perkins's line); Tönnis grade II refers to the ossification center of the capital epiphysis is lateral to the Perkin's line, but below the superolateral margin of the acetabulum (SMA-line). Tönnis grade III refers to the ossification center is at the level of the center the superolateral margin of the acetabulum. Tönnis grade IV refers to the ossification center is above the superolateral margin of the acetabulum acetabulum (2). For children with high displacement like Tönnis grade III and IV DDH, especially those >2 years, a femoral shortening osteotomy was usually required to prevent excessive pressure on the femoral head, or correct excessive femoral anteversion, to improve the stability of the hip and decrease the incidence of avascular necrosis (AVN) (3–5). Limb length discrepancy (LLD) was a common postoperative complication in children with DDH (6, 7). A pelvic or femoral shortening osteotomy, excessive growth after the femoral osteotomy, poor functional recovery of the hip joint and lower limb after the operation, and other factors may lead to long-term limb length inequality. An increasing number of studies show that LLD is harmful to patients, and may cause low back pain, hip and knee pain, stress fractures and scoliosis (7–9). Many studies have analyzed the related risk factors of overgrowth after traumatic fracture of the lower limb in children, including gender, age, fracture location, and operation mode (10–13). Overgrowth is caused by the destruction of periosteum integrity, the reduction of pressure on the growth plate, and an increase in blood supply (14, 15). However, there was no research on the causes or risk factors of postoperative LLD in children with DDH, especially on overgrowth of the affected limbs after combined femoral shortening osteotomy. Therefore, we aimed to analyze children with unilateral DDH who underwent proximal femoral shortening osteotomy, to explore the related risk factors of overgrowth and LLD.

## Materials and methods

### Study population and data collection

The study protocol was approved by the institutional review board of our institution. The age, sex, side, operation duration, operation method, femoral shortening osteotomy length, femoral length at follow-up, and anatomical length of both lower limbs were recorded. The inclusion criteria were as follows: (1) children aged 2–12 years; (2) DDH diagnosed by x-ray and CT examination, and without any other musculoskeletal diseases; (3) unilateral onset, without any previous treatment such as closed and open reduction before the operation, and no second operation on the same side at a later date; (4) regular follow-up, with full-length lower extremity weight-bearing radiographs after the operation, and a follow-up time of more than 24 months; (5) preoperative examination showing no contraindications; (6) full informed consent provided by each child and legal guardian.

Through the examination of medical records, information such as gender, age, side of limb, operation mode, operation duration, osteotomy length and follow-up time was obtained. All the children were treated with a Salter pelvic and Pemberton osteotomy combined with femoral shortening osteotomy. According to the match between the femoral head and acetabulum, and where the acetabulum covered the femoral head, Salter pelvic osteotomy and Pemberton osteotomy were selected (16, 17), respectively. At the same time, a femoral shortening osteotomy was selected according to the difficulty of femoral head reduction and the pressure between the acetabulum and the femoral head after reduction, and the length of shortening was evaluated.

### Surgical procedure

**Figure 1:**
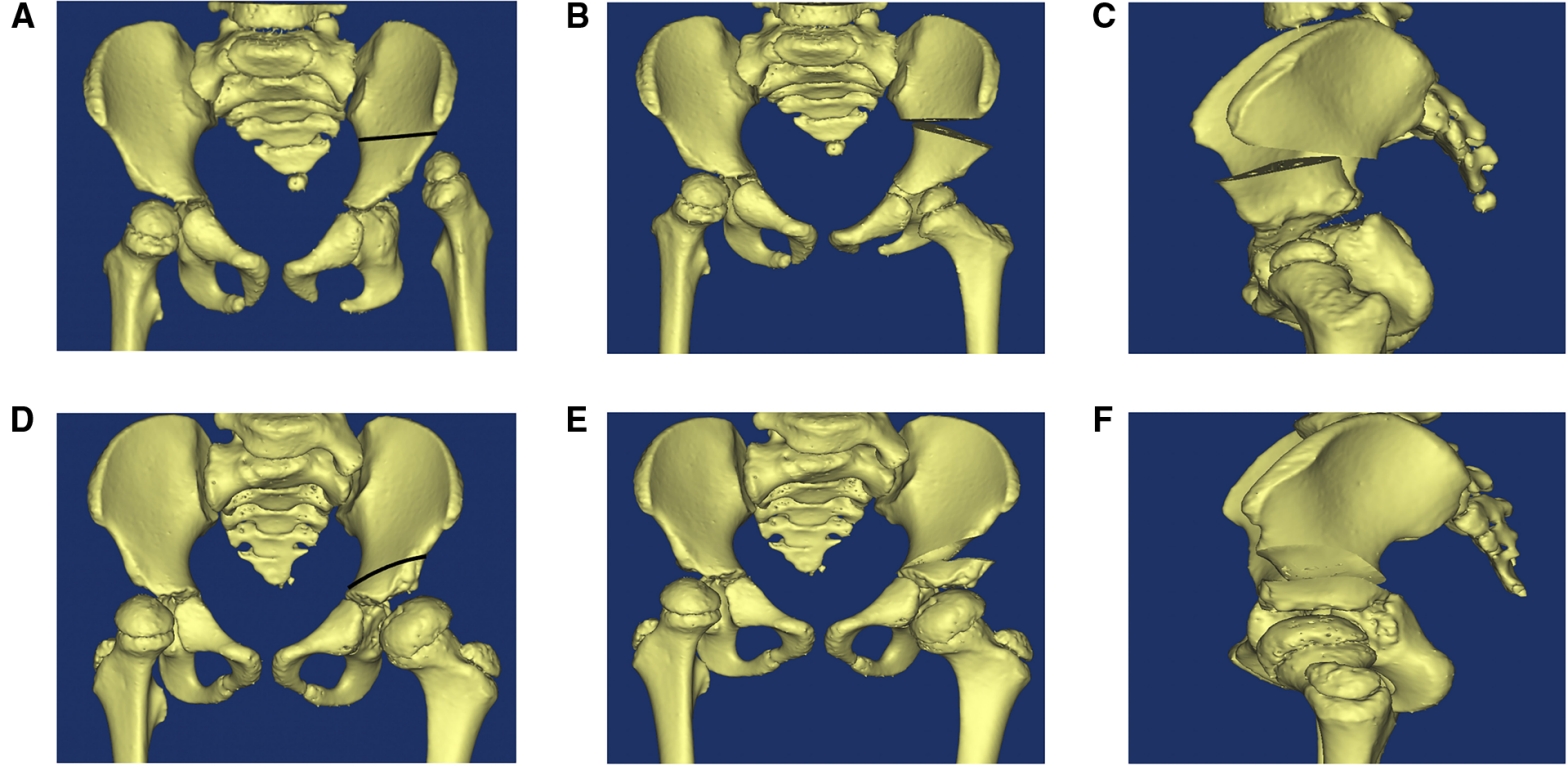
Salter pelvic osteotomy and Pemberton osteotomy. (**A**) The osteotomy line of Salter pelvic osteotomy (blank line). (**B**) Anteroposterior view of pelvic after salter osteotomy. (**C**) Lateral view of pelvic after salter osteotomy. (**D**) The osteotomy line of Pemberton osteotomy (blank line). (**E**) Anteroposterior view of pelvic after pemberton osteotomy. (**F**) Lateral view of pelvic after pemberton osteotomy.

After induction of general anesthesia, patients were placed in the supine position on a radiolucent table with a bump placed under the lumbosacral spine to provide a 30-degree elevation of the ipsilateral hip. A “bikini” oblique incision was made and then the hip joint was exposed by capsulotomy with Type T incision. An additional lateral femoral straight incision was made for femoral shortening osteotomy. Plate and screws were used for internal fixation after femoral shortening osteotomy. Then a Salter osteotomy or Pemberton osteotomy were selected according to the relationship between the acetabulum and the femoral head (Figure 1). Pemberton osteotomy: An iliac cortical lateral osteotomy was performed 1 cm above the hip joint capsule. The osteotomy began at the level of the anterior inferior iliac spine and extended to the triradiate cartilage. The distal fragment was then pressed down and a wedge of bone was resected and inserted into the resulting space. The joint capsule was mobilized to the level of the acetabulum, the femoral head was reduced, hemostasis was achieved, and the lower limb and hip abductor stent were applied for fixation. Salter pelvic osteotomy: A vertical osteotomy was performed between the upper and lower iliac spines and the ischial notch. The distal fragment was then rotated forward, outward and downward, and filled with triangular autogenous iliac bone. Followed by fixing the two pieces using three Kirschner wires. Finally, we repaired the joint capsule and closed the incision. Hip braces were applied after the operation.

### Imaging evaluation

**Figure 2:**
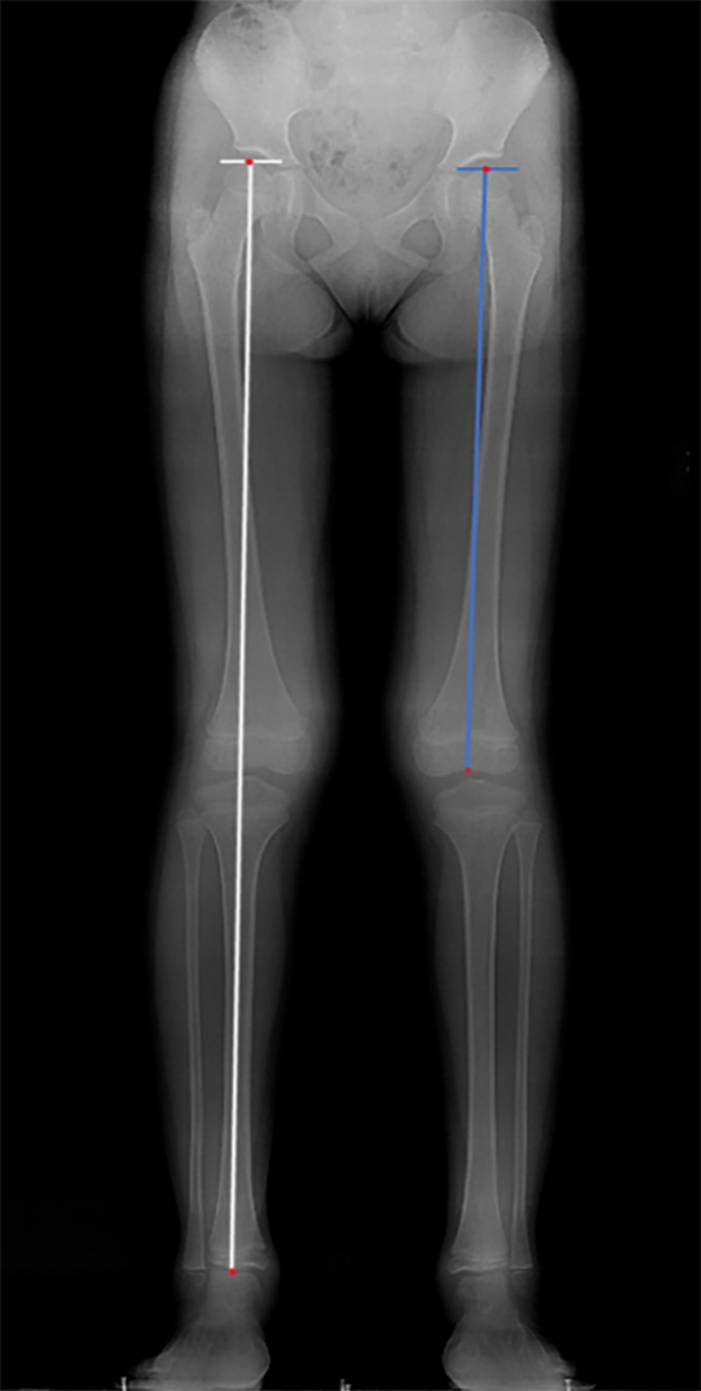
Measurement of whole lower limb and femur length. white line: whole lower limb length; blue line: femur length.

Postoperative imaging protocol included postoperative pelvic radiographs (including the proximal femur) taken at 1 day, 1 month, 2 months, and 3 months after surgery, as well as full-length lower extremity weight-bearing radiographs taken at 6 months, 12 months, and annually thereafter. We have used the following formula to calculate overgrowth: (affected femur length at final follow-up – healthy femur length at final follow-up + intraoperative femoral osteotomy length). At the last follow-up, the femoral length and the whole limb length (WLL) were measured (18, 19). The femoral length was regarded as the distance from the top of the femoral head to the farthest point in the intercondylar fossa of the distal femur; the WLL, the distance from the top of the femoral head to the center of the tibial plafond (Figure 2); and LLD, the difference between the total limb lengths of two sides.

### Statistical analysis

SPSS 17.0 software (SPSS Inc., Chicago, Illinois, USA) was used for statistical analysis, independent sample T test was used for comparisons between groups, and chi-square test or Fisher's exact test was used in the analysis of contingency data. The risk factors affecting overgrowth ≥1 cm and LLD ≥ 1 cm were tested by binary logistic regression analysis. *P *< 0.05 was regarded as statistically significant.

## Results

**Figure 3:**
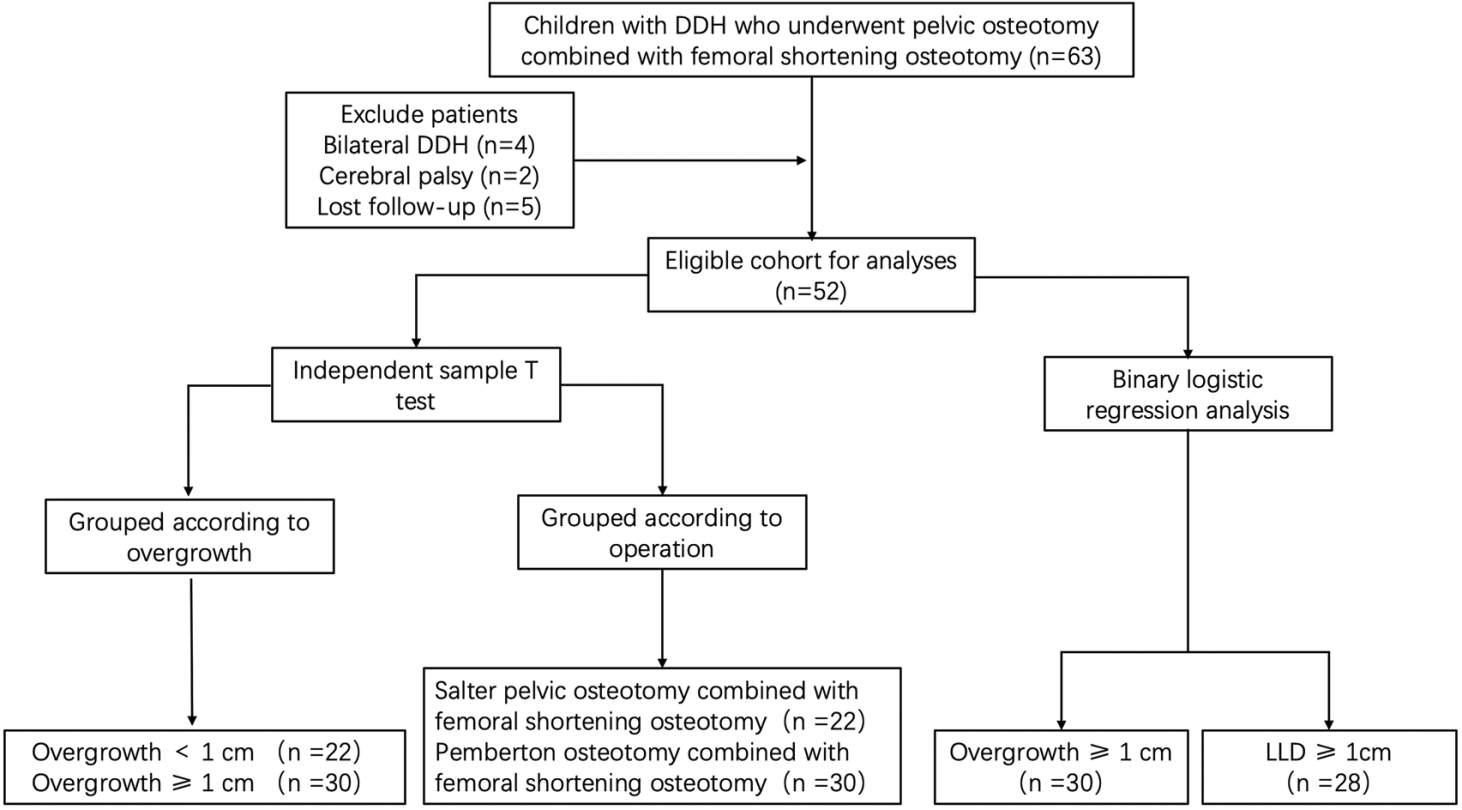
A flowchart of patient’s selection and study design. DDH: developmental dysplasia of the hip; LLD: limb length discrepancy.

In our research center, a total of 63 cases of pelvic osteotomy combined with femoral shortening osteotomy were completed from January 2016 to April 2018. However, 2 cases of cerebral palsy, 4 cases of bilateral DDH, and 5 cases of missing full-length lower extremity weight-bearing radiographs at the last follow-up were excluded according to our inclusion and exclusion criteria. Finally, a total of 52 patients were included in the study (Figure 3). The average age of the children at the time of operation was 5.00 ± 2.48 years old, including seven males and 45 females. There were 39 cases on the left side and 13 cases on the right side.

**Figure 4:**
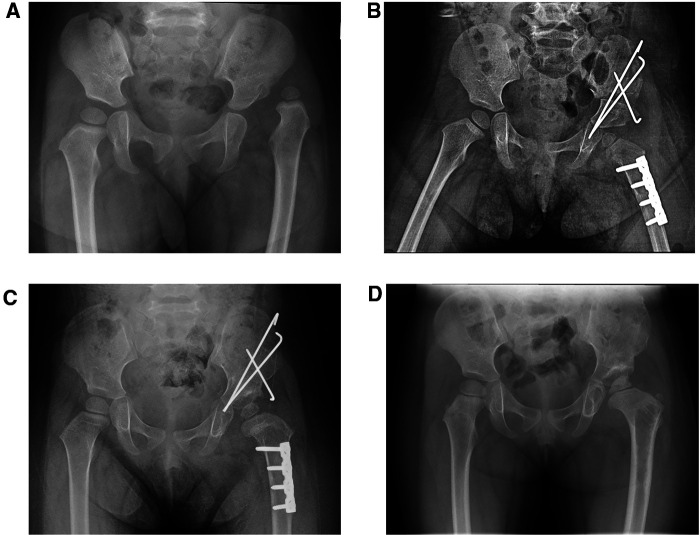
A girl, aged 2 years and 4 months, with Tönnis III dislocation of her left hip, underwent a Salter pelvic osteotomy combined with femoral shortening osteotomy. (**A**) Preoperative hip anteroposterior (AP) radiograph: left hip dislocation. (**B**) AP radiograph obtained one month after the Salter pelvic osteotomy combined with femoral shortening osteotomy. The length of the femur osteotomy was 1.5 cm. (**C**) AP radiograph obtained six months after the Salter pelvic osteotomy combined with femoral shortening osteotomy. (**D**) AP radiograph obtained 14 months after the Salter pelvic osteotomy combined with femoral shortening osteotomy and 6 months after removal of internal fixation.

**Figure 5:**
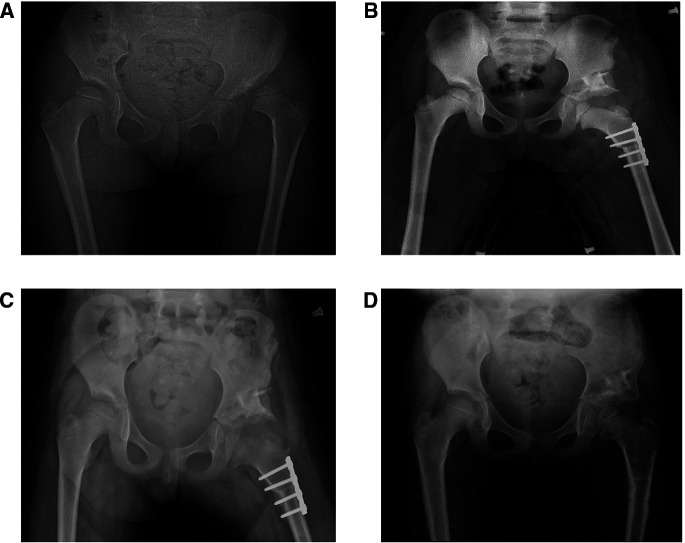
A girl, aged 6 years and 8 months, with Tönnis II dislocation of her left hip, underwent a Pemberton osteotomy combined with femoral shortening osteotomy. (**A**) Preoperative hip anteroposterior (AP) radiograph: left hip dislocation. (**B**) AP radiograph obtained three days after the Pemberton osteotomy combined with femoral shortening osteotomy; the length of the femoral osteotomy is 2 cm. (**C**) AP radiograph obtained 1.5 months after the Pemberton osteotomy combined with femoral shortening osteotomy. (**D**) AP radiograph obtained 12 months after the Pemberton osteotomy combined with femoral shortening osteotomy and 2 months after removal of internal fixation.

**Figure 6:**
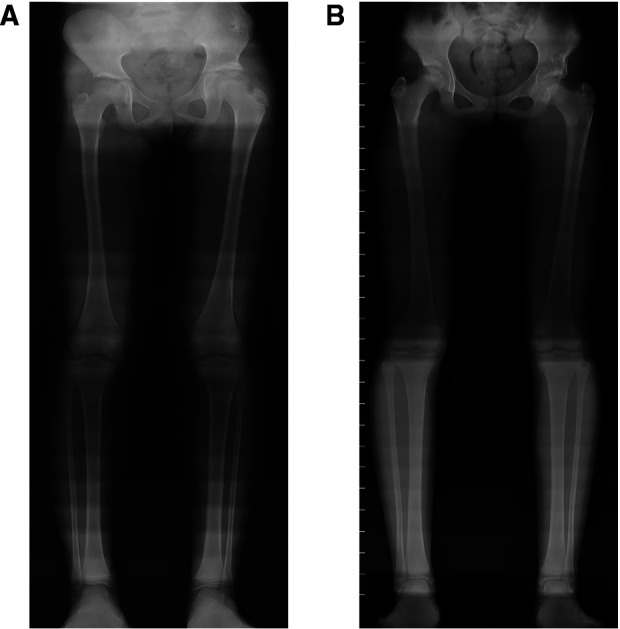
Two cases of LLD after pelvic osteotomy combined with femoral shortening osteotomy (**A**) full-length lower extremity weight-bearing radiographs obtained 50 months after the Salter pelvic osteotomy combined with femoral shortening osteotomy. The length of left and right femur was 350 mm and 343 mm respectively. The whole length of the left and right lower limb was 647 mm and 638 mm respectively. (**B**) Full-length lower extremity weight-bearing radiographs obtained 47 months after the Pemberton osteotomy combined with femoral shortening osteotomy. The length of left and right femur was 378 mm and 391 mm respectively. The whole length of the left and right lower limb was 706 mm and 721 mm respectively.

Twenty-two children underwent Salter pelvic osteotomy combined with femoral shortening osteotomy (Figure 4), and 30 underwent Pemberton osteotomy (Figure 5) combined with femoral shortening osteotomy. At the last follow-up, full-length lower extremity weight-bearing radiographs were taken and the length of lower limbs was measured (Figure 6). The average operation duration was 3.23 ± 1.09 hours, the average femoral osteotomy length was 1.26 ± 0.67 cm, and the average follow-up time was 45.85 ± 6.22 months. The average overgrowth of all children was 1.20 ± 0.82 cm, of which 30 cases had overgrowth of the femur ≥1 cm, and 22 cases had overgrowth of the femur <1 cm (Table 1). In addition, avascular necrosis (AVN) was observed in four cases, two of which occurred following Salter pelvic osteotomy and the other two following Pemberton osteotomy.

**Table 1:** Summary of patient demographics.

	Mean ± standard deviation
Gender (males/females)	7/45
Side (left/right)	39/13
Age (years)	5.00 ± 2.48
Surgery type (surgery 1/surgery 2)	22/30
Operation duration (hours)	3.23 ± 1.09
Osteotomy length (cm)	1.26 ± 0.67
Follow-up time (months)	45.85 ± 6.22
Overgrowth (cm)	1.20 ± 0.82

Surgery 1: Salter pelvic osteotomy combined with femoral shortening osteotomy. Surgery 2: Pemberton osteotomy of pelvis combined with femoral shortening osteotomy.

There were statistical differences in age (*p *< 0.001) and operation duration (*p *= 0.010) between the two groups with femoral overgrowth <1 cm and ≥1 cm, while gender, length of osteotomy, and operation mode were not significant (Table 2).

**Table 2:** Comparison of variables between the two groups of patients with femoral overgrowth <1 cm and ≥1 cm.

	Overgrowth <1 cm (*N* = 22)	Overgrowth ≥1 cm (*N* = 30)	*p-*value
Gender (males/females)	1/21	6/24	0.107
Side (left/right)	16/6	23/7	0.746
Age (years)	7.34 ± 1.76	3.29 ± 1.16	<0.001
Operation duration (hours)	3.69 ± 0.93	2.90 ± 1.09	0.010
Length of osteotomy (cm)	1.39 ± 0.63	1.17 ± 0.69	0.259
Surgery type (surgery 1/ surgery 2)	8/14	14/16	0.458
Follow-up time (months)	46.64 ± 6.78	45.27 ± 5.83	0.438

Surgery 1: Salter pelvic osteotomy combined with femoral shortening osteotomy. Surgery 2: Pemberton osteotomy of pelvis combined with femoral shortening osteotomy.

There was a statistical difference in operation duration between surgery 1 and surgery 2 (*p *< 0.001), but there was no statistical difference in sex, side, age, femoral osteotomy length, follow-up time, and femoral overgrowth between the two groups (Table 3).

**Table 3:** Comparison of variables between the two groups of children who underwent surgery 1 and surgery 2.

	**Surgery** 1 (*n* = 22)	**Surgery** 2 (*n* = 30)	*p-*value
Gender (males/females)	1/21	6/24	0.107
Side (left/right)	17/5	22/8	0.746
Age (years)	4.67 ± 2.56	5.39 ± 2.38	0.186
Operation duration (hours)	2.60 ± 0.77	3.69 ± 1.07	<0.001
Osteotomy length (cm)	1.18 ± 0.64	1.32 ± 0.69	0.455
Follow-up time (months)	45.64 ± 6.03	46.00 ± 6.46	0.837
Overgrowth (cm)	1.35 ± 0.80	1.08 ± 0.83	0.251

Surgery 1: Salter pelvic osteotomy combined with femoral shortening osteotomy. Surgery 2: Pemberton osteotomy of pelvis combined with femoral shortening osteotomy.

In addition, age (*p *< 0.001) was an independent influencing factor of femoral overgrowth after pelvic osteotomy and femoral shortening osteotomy in children with unilateral DDH, and the incidence of femoral overgrowth ≥1 cm will increase by 2.56 times every year of age decrease. The operation duration, sex, side, length of femoral osteotomy, and operation mode were not independent influencing factors of femoral overgrowth ≥1 cm (Table 4).

**Table 4:** Analysis of risk factors of femoral overgrowth ≥1 cm after DDH operations.

Variable	*B*	Exp (B)	95% Confidence interval of Exp (B)	*p-*value
Age (years)	−1.268	0.281	0.142–0.560	<0.001
Gender (males/females)	−1.426	0.240	0.145–11.191	0.549
Operation duration (hours)	0.225	1.253	0.355–4.419	0.726
Osteotomy length (cm)	0.309	1.361	0.262–7.064	0.713
Surgery type (surgery 1 /surgery 2)	−3.107	0.450	0.001–2.304	0.122
LLD (cm)	−2.766	0.063	0.004–1.109	0.590

Surgery 1: Salter pelvic osteotomy combined with femoral shortening osteotomy. Surgery 2: Pemberton osteotomy of pelvis combined with femoral shortening osteotomy. DDH, developmental dysplasia of the hip; LLD, limb length discrepancy.

Age (*p *= 0.008) was a risk factor for LLD in children with unilateral DDH after pelvic osteotomy and femoral shortening osteotomy, and the incidence of LLD ≥ 1 cm will increase by 1.06 times with each age increase. Other variables were not independent influencing factors of LLD ≥ 1 cm (Table 5).

**Table 5:** Risk factor analysis of children with LLD ≥ 1 cm after DDH operation.

Variable	*B*	Exp (B)	95% Confidence interval of Exp (B)	*p-*value
Age (years)	0.724	2.062	1.210–3.514	0.008
Gender (males/females)	1.799	6.042	0.698–52.340	0.102
Operation duration (hours)	−0.252	0.777	0.337–1.793	0.555
Osteotomy length (cm)	0.600	1.822	0.597–5.564	0.292
Surgery type (surgery 1 /surgery 2)	−0.931	0.394	0.074–2.108	0.276
Overgrowth (cm)	1.315	3.726	0.803–17.293	0.093

Surgery 1: Salter pelvic osteotomy combined with femoral shortening osteotomy. Surgery 2: Pemberton osteotomy of pelvis combined with femoral shortening osteotomy. DDH, developmental dysplasia of the hip; LLD, limb length discrepancy

## Discussion

There were statistical differences in age and operation duration between the two groups with femoral overgrowth <1 cm and ≥1 cm. There was also a statistical difference in operation duration between these groups. Age is an independent influencing factor of femoral overgrowth in children with unilateral DDH after pelvic osteotomy combined with femoral shortening osteotomy, and a risk factor of LLD in children with unilateral DDH after pelvic osteotomy combined with femoral shortening osteotomy. The causes of overgrowth in children after trauma are complex, but one possible explanation is related to the integrity of the periosteum. According to Hueter-Volkmann's law, increased pressure on the growth plate can inhibit bone growth, while decreased pressure can lead to accelerated growth. This idea was first proposed by Ollier in 1873 (20), who observed that overgrowth after fracture was often associated with damage to the periosteum. Subsequent research has supported this concept, and animal models have been developed to further investigate the relationship between periosteum and overgrowth. A study by Halanski et al. (14) compared the effects of different types of periosteum incisions on overgrowth, and found that circular periosteum incisions were more likely to lead to overgrowth than longitudinal incisions. The study found that after 2–8 weeks, the proliferation and differentiation of growth plate cells in the distal tibia were more active than in the contralateral tibia, suggesting that traction along the longitudinal axis of the long bone may effectively limit overgrowth. This idea was supported by the findings of Bertram et al. (21), who measured the periosteal tension at different positions and created a strain-strain curve. They observed that the periosteal tension increased by 1 Newton per millimeter from the proximal to the distal end of the growth plate, and concluded that the tighter the periosteum adhesion, the greater the pressure generated at both ends, resulting in the greatest pressure at the growth plate and metaphysis. The study design implemented was femoral osteotomy was done during operation, and femoral periosteum was protected as much as possible by transverse osteotomy, resulting in the destruction of periosteum integrity. This ultimately resulted in the reduction of pressure on the growth plates at both ends of the femur, thereby leading to postoperative overgrowth.

Femoral shortening osteotomy is usually combined with acetabuloplasty (22) to treat children with high dislocation rates of the hip joint or high compression of the femoral head when the femoral head is reset into the acetabulum. Femoral shortening osteotomy is a surgical procedure that involves altering the anatomical length of the femur. Additionally, the osteotomy process itself can result in a fracture of the femoral cortex, which has been found to be associated with an increased risk of post-surgical excessive growth. This combination of factors has been identified as a significant contributor to the development of LLD in children with DDH following pelvic osteotomy (6). However, the LLD is harmful to children and may cause lower back pain, stress fractures, hip and knee arthritis, and other complications (8, 23, 24). In recent years, many studies have shown that even mild LLD can also lead to biomechanical changes of the lower limbs in children (25, 26). ng. Therefore, it is very important to determine the influencing factors of LLD in children with DDH after pelvic osteotomy combined with femoral shortening osteotomy. This study shows that the operative age of children with DDH was significantly related to LLD.

Many studies have shown that overgrowth occurs after lower limb fracture in children, and the related risk factors have been analyzed. In studies of the influencing factor of age on overgrowth of the lower limbs in children, Sulaiman et al. (27) studied 15 children (aged ranged from 8 to 14 years old) with femoral shaft fractures, including 12 boys and three girls. The overgrowth range of all injured femurs was 0.1–2.0 cm, with an average of 1.15 cm. The results show that there was a strong correlation between age and femoral overgrowth (R = −0.94, *p *< 0.05). Stilli et al. (28) conducted a multicenter study, which included 1,162 children with femoral shaft fractures and 822 children with tibial shaft fractures, with an average follow-up time of 6.6 years. The study showed that the average overgrowth of femoral and tibial fractures was 9.2 and 5.7 mm, respectively. Children under five years of age had more overgrowth. However, Kim et al. (29) in a study of the relationship between femoral shaft fracture and overgrowth in children, showed that there was no statistical difference between the amount of overgrowth of the femur and age. The results of our study are similar to those of Sulaiman et al. and Stilli et al. In this study, there was a statistical difference (*p* = 0.010) between the two groups of femoral overgrowth <1 cm and femoral overgrowth ≥1 cm, and the incidence of femoral overgrowth ≥1 cm will increase by 2.56 times with each year of age reduction.

Clement and Colton (30) studied 29 children with femoral fractures. The results showed that the overgrowth ability of boys was stronger than that of girls, and the gender of the patients was the most important factor affecting overgrowth. Choi et al. (31) confirmed this by including 104 children under 14 years old with tibial fractures in a study, and the effects of age, sex, fracture type, and other factors on overgrowth after fracture were analyzed; the risk of an LLD ≥ 1 cm in boys was 7.4 times higher than in girls, and the risk of overgrowth ≥1 cm was 5.4 higher than in girls. The authors assumed that the stimulation of bone growth by congestion had a greater impact on the growth plates of the boys. In addition, because boys are more active, they are more prone to high-energy injuries. However, Stephens et al. (32) believed gender was not a factor affecting overgrowth in children. At present, there was still a lack of clinical research on the influence of sex on overgrowth in this population. Although our research object was not children with femoral fractures, all children in this study underwent femoral shortening osteotomies. We believe that the destruction of femoral continuity caused by surgical intervention is similar to that of femoral shaft fractures, and we found that there was no statistical difference in femoral overgrowth between boys and girls. Despite the incidence rate of girls with DDH was much higher than that of boys, there were fewer boys in this study, which does not reflect the relationship between gender and overgrowth.

In addition, in this study, according to the different hip joint operation mode, the children were divided into two groups, and it was found that there was no statistical difference between the two different operation mode on femoral overgrowth. At present, there is no report on the effect of different pelvic osteotomies on the overgrowth of the lower limbs in children. The results of this study may indicate that acetabuloplasty for DDH was not an important factor causing femoral overgrowth in children, but the true cause of femoral overgrowth was femoral osteotomy. There was a statistical difference in the operation duration between the two methods, and there was also a statistical difference in the operation duration between the two groups of femoral overgrowth <1 and ≥1 cm. The reason for the longer surgery duration of Pemberton osteotomy was that older children have stronger soft tissue, making the surgery more difficult. Additionally, the complexity of the Pemberton osteotomy procedure, which requires multiple x-ray fluoroscopies during the operation, may contribute to the increased surgery duration compared to the Salter pelvic osteotomy. There were 24 children with an LLD ≥ 1 cm, and it was found that age was significantly related to LLD during postoperative follow-up, and the incidence of LLD would increase by 1.06 times with each year of age increase. The length of the affected femur was shortened, so, although the affected limb was overgrown during the postoperative follow-up, if the overgrowth was less than the femoral shortening, the affected limb would be shortened. On the contrary, this will lead to the lengthening of the affected limb. The results of this experiment may be due to the weak overgrowth ability of the older children, which leads to overgrowth of the affected limb after the osteotomy, which cannot make up for the shortening of the femur, resulting in the shortening of the affected limb. However, younger children have stronger overgrowth ability, and better overgrowth of affected limbs can make up for the shortened femoral osteotomy length, which leads to smaller LLD in younger children.

This study had some limitations. First, our minimum follow-up time was 35 months. Our results show that the follow-up time has nothing to do with the overgrowth of femur in children after surgery, which requires further long-term follow-up study. Second, we did not take a full-length radiographic film of both lower limbs before the operation, and assumed that the lengths of the lower limbs were equal. Third, our sample size was small and was not estimated because this study has a retrospective design. We could not determine the risk factors of LLDs ≥ 2 cm, because only 11 patients had such. According to the calculation of the sample size, further large cohort studies are needed to provide meaningful clinical information.

In conclusion, we prove that overgrowth and LLD after pelvic osteotomy combined with femoral shortening osteotomy in children with DDH was significantly related to age. There was no significant difference between femoral overgrowth and different pelvic osteotomy type. Future research should increase the sample size, and extend the follow-up time to explore the rule of overgrowth after femoral osteotomy. Surgeons should consider age, dislocation height, pelvic osteotomy method, overgrowth length and other factors when conduct femoral shortening osteotomy for DDH patients.

## Data Availability

The original contributions presented in the study are included in the article/Supplementary Material, further inquiries can be directed to the corresponding author/s.
